# A model-based assessment of social isolation practices for COVID-19 outbreak response in residential care facilities

**DOI:** 10.1186/s12879-024-09788-x

**Published:** 2024-08-29

**Authors:** Cameron Zachreson, Ruarai Tobin, Camelia Walker, Eamon Conway, Freya M. Shearer, Jodie McVernon, Nicholas Geard

**Affiliations:** 1 School of Computing and Information Systems, The University of Melbourne, Parkville, Victoria Australia; 2 Centre for Epidemiology and Biostatistics, Melbourne School of Population and Global Health, The University of Melbourne, Parkville, Victoria Australia; 3 School of Mathematics and Statistics, The University of Melbourne, Parkville, Victoria Australia; 4 The Walter and Eliza Hall Institute, Parkville, Victoria Australia; 5 Victorian Infectious Disease Reference Laboratory Epidemiology Unit, The Royal Melbourne Hospital at the Peter Doherty Institute for Infection and Immunity, Melbourne, VIC Australia; 6 Department of Infectious Diseases, The University of Melbourne at the Peter Doherty Institute for Infection and Immunity, Melbourne, VIC Australia

**Keywords:** Residential aged care, Nursing homes, Long-term care, Agent-based model, COVID-19, Outbreak response, Social isolation, Nonpharmaceutical intervention, Infectious disease dynamics, Contact network

## Abstract

**Background:**

Residential aged-care facilities (RACFs, also called long-term care facilities, aged care homes, or nursing homes) have elevated risks of respiratory infection outbreaks and associated disease burden. During the COVID-19 pandemic, social isolation policies were commonly used in these facilities to prevent and mitigate outbreaks. We refer specifically to general isolation policies that were intended to reduce contact between residents, without regard to confirmed infection status. Such policies are controversial because of their association with adverse mental and physical health indicators and there is a lack of modelling that assesses their effectiveness.

**Methods:**

In consultation with the Australian Government Department of Health and Aged Care, we developed an agent-based model of COVID-19 transmission in a structured population, intended to represent the salient characteristics of a residential care environment. Using our model, we generated stochastic ensembles of simulated outbreaks and compared summary statistics of outbreaks simulated under different mitigation conditions. Our study focuses on the marginal impact of general isolation (reducing social contact between residents), regardless of confirmed infection. For a realistic assessment, our model included other generic interventions consistent with the Australian Government’s recommendations released during the COVID-19 pandemic: isolation of confirmed resident cases, furlough (mandatory paid leave) of staff members with confirmed infection, and deployment of personal protective equipment (PPE) after outbreak declaration.

**Results:**

In the absence of any asymptomatic screening, general isolation of residents to their rooms reduced median cumulative cases by approximately 27%. However, when conducted concurrently with asymptomatic screening and isolation of confirmed cases, general isolation reduced the median number of cumulative infections by only 12% in our simulations.

**Conclusions:**

Under realistic sets of assumptions, our simulations showed that general isolation of residents did not provide substantial benefits beyond those achieved through screening, isolation of confirmed cases, and deployment of PPE. Our results also highlight the importance of effective case isolation, and indicate that asymptomatic screening of residents and staff may be warranted, especially if importation risk from the outside community is high. Our conclusions are sensitive to assumptions about the proportion of total contacts in a facility accounted for by casual interactions between residents.

**Supplementary Information:**

The online version contains supplementary material available at 10.1186/s12879-024-09788-x.

## Background

Residential aged-care facilities (RACFs, also called long-term care facilities, aged care homes, or nursing homes) have elevated risks of respiratory infection outbreaks and associated disease burden. Heightened risk arises due to importation of pathogens by visitors and staff, close and prolonged contact among residents (occupants of the facility) and staff members, and the demographic and health profiles of residents. These intersecting factors produce scenarios in which outbreaks are difficult to mitigate and carry disproportionate consequences in terms of medical impact. Simultaneously, the physical infrastructure, insecure workforce, high health needs of residents, and particular social requirements of RACFs impose limitations on the types of control measures that can be enacted without compromising the mental health of residents [1]. In contrast with other types of healthcare settings, RACFs feature long-term stays (multiple years [2]), frequent social interactions between residents, and a large part-time workforce with variable levels of medical training [3].

Modelling efforts made early during the COVID-19 pandemic did not emphasize the confluence of risk factors present in aged-care environments (e.g., high prevalence of comorbidity and limited infection control capacity) that would later be acknowledged as responsible for large numbers of preventable deaths [4]. This led to a substantial and ongoing global research effort applying infectious disease modelling to outbreak detection, response, and prevention in residential care scenarios [5–11].

While modelling improved substantially following the acknowledgement of the disproportionate clinical significance of COVID-19 outbreaks in aged care, the strength of evidence for intervention effectiveness from observational and modelling studies has still been assessed as generally weak [12, 13]. This has so far limited the quality of information available to policy makers faced with challenging and complex decisions about COVID-19 response measures in aged care.

In particular, there is a lack of modelling work that specifically assesses the effectiveness of general isolation conditions applied to resident populations, despite these being possibly the most controversial policies implemented across the sector globally. By “general isolation” we refer specifically to policies that were intended to reduce contact between residents, without regard to confirmed infection status. These include, for example, shutting down shared meal facilities and closing social spaces at facilities, or encouraging residents to stay in their rooms. Around the world, though such policies were not consistently included in government recommendations, many research articles, clinical reports, interviews, media reports, and informal testimony agree that facilities implemented general isolation measures in attempts to reduce the impact of ongoing outbreaks [14–19].

Here we apply an agent-based model of outbreak mitigation in aged care, to investigate the effectiveness of such policies, and assess the conditions under which they may be justified. For a realistic assessment, our model includes other generic interventions consistent with the Australian Government’s recommendations released during the COVID-19 pandemic: isolation of confirmed resident cases, furlough (mandatory paid leave) of staff with confirmed infection, and deployment of personal protective equipment (PPE) after outbreak declaration [20].

## Methods

### Overview

In consultation with the Australian Government Department of Health and Aged Care, we developed an agent-based model of COVID-19 transmission in a structured population, building upon and modifying earlier modelling frameworks used to assess international arrival and quarantine pathways [21]. In general, structured populations feature spatial and temporal clustering in contact patterns between individuals determined by, for example, heterogeneous but persistent dwelling locations and activity schedules. For such populations, homogeneous assumptions about contact patterns are not appropriate for detailed simulations of infectious disease transmission dynamics. To capture these important structural properties, key features of the model include detailed representation of:

- Facility characteristics, including resident and staff numbers;
- Contact patterns among staff and residents, incorporating details of staff scheduling;
- Infection dynamics, including time-varying infectiousness and test sensitivity;
- Screening and response strategies.

To capture the limitations of early detection, we implemented realistic screening and response strategies. These were based on those set out in the Australian Government Department of Health document “COVID-19 Outbreaks in Residential Care Facilities, Communicable Disease Network Australia, National Guidelines for the Prevention, Control and Public Health Management of COVID-19 Outbreaks in Residential Care Facilities” dated February 15th, 2022 [20]. Full details of the model implementation and assumptions are provided in the Supporting Information.

### Facility structure

**Fig. 1:**
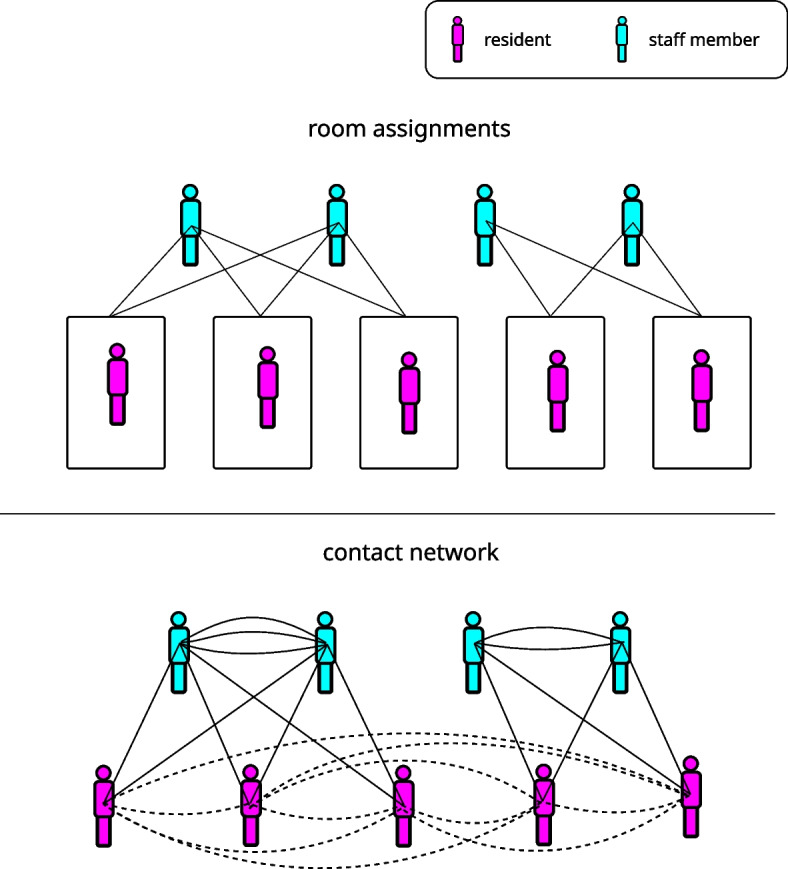
To generate a structured contact network, residents are assigned to rooms in which they live and staff members are assigned to sets of rooms that they service. This is done to ensure that each room is serviced by the same number of staff. Potential contacts are then created between any two individuals who are assigned to the same room. This creates a network structure from which contacts are sampled during simulation of transmission dynamics. In the contact network depicted here, solid lines represent edges derived from room assignments while dashed lines represent potential casual contacts between residents

Briefly, a facility is modelled as a static population of residents, allocated to single-occupancy rooms. For this study, we simulated a facility with 121 staff and 88 residents, which is a typical size for an Australian RACF [22]. Staff attend a facility according to a weekly roster, with each staff member working five, three, or two days per week which accounts for a total of 73.8 full-time equivalent (FTE) staff (in Australia, 1.0 FTE corresponds to 5 full-time shifts in one week). Staff members are allocated to a set of rooms housing residents they will visit during the days they attend a facility. These allocations are used to generate a network of potential contacts, from which potentially infectious contact events are randomly sampled (Fig. 1). In addition to these structured contacts, random contacts between residents in different rooms are included separately to allow for interactions mediated by communal areas and group activities that are not explicitly simulated in the facility model. Infectious contacts are subject to a probabilistic transmission process that depends on the time since the transmitting case was infected and any mitigation measures in place (see Supporting Information).

### Within-host model of infection

Infection proceeds through an incubation period, followed by symptom expression and eventual recovery (see the Supporting Information for more details on the model of individual infectiousness trajectories). Each infected individual is potentially infectious from the moment of exposure, through the incubation period and the duration of symptoms. Infectiousness increases until approximately the time of symptom onset, and then declines until recovery. We assume that, on average, 33% of infections never express symptoms and that symptom expression does not alter infectiousness given contact with someone who is susceptible. That is, the time-dependent trajectory of infectiousness is identical for symptomatic and asymptomatic infections. However, case detection following symptom onset may alter contact patterns due to infection control procedures, see below. The assumed asymptomatic fraction of 33% is consistent with global estimates, however, we do not account for heterogeneity by age which could slightly increase the likelihood of case detection in residents relative to staff members [23–26].

### Baseline transmission dynamics

**Fig. 2:**
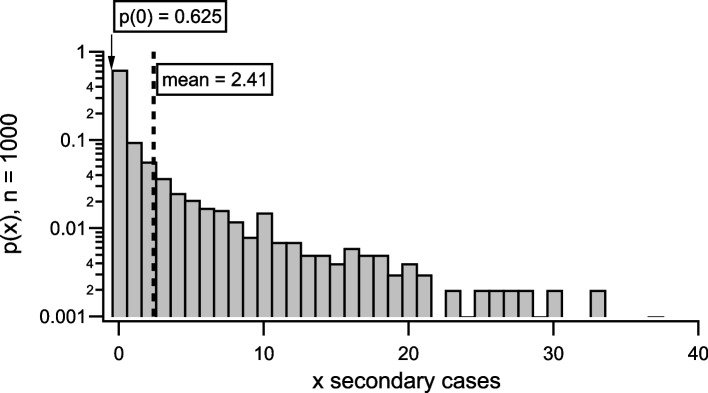
Index case secondary case distribution for 1000 realisations

In line with observed SARS-CoV-2 transmission dynamics, the model includes overdispersion of case infectiousness, in which approximately 60% of index cases produce no secondary cases, while approximately 10% of index cases produce more than ten secondary cases (Fig. 2) [27, 28].

We calibrated the disease transmission model to produce a reproductive ratio of $R_0 \approx 2.4$, to simulate a scenario consistent with SARS-CoV-2 transmission in populations with modest immune-derived protection. These conditions correspond approximately to early estimates of the effective reproduction number of the Omicron variant [29]. Further, the reproductive ratio we selected is broadly consistent with the assumptions used in previous modelling studies [5, 8, 30]. See the Supporting Information for more details on calibration.

### Initialisation

Each simulation commences with a single index case and runs until there are no infections remaining in a facility or, in the case that an outbreak has been declared, the outbreak is declared to be over. Index cases are selected at random from the combined population of staff and residents. We note that this initialisation means that we are not simulating continuous importation of cases from the community. This choice excludes from the scope of our study any assessment on the role of visitation restrictions, community prevalence, or the behaviour of staff members outside of the facility. Rather, our model focuses on the transmission dynamics produced by single index cases.

### Outbreak detection and response

**Fig. 3:**
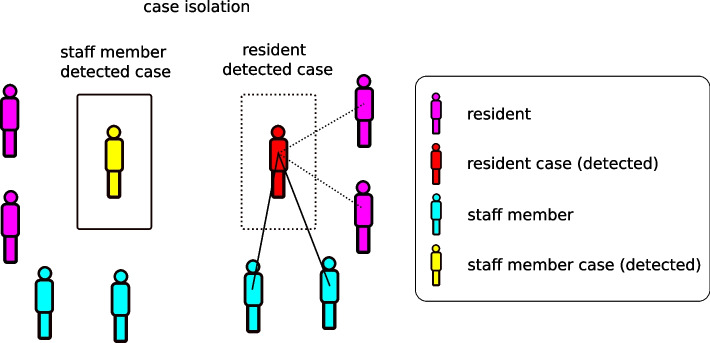
Schematic of case isolation procedure after detection through a positive test result. Residents remain in contact with staff members at the same frequency regardless of case isolation, but have reduced levels of contact with other residents (contact frequency with residents in different rooms reduced by 90%). Isolated staff members are completely removed from contact (furloughed, not present at the facility)

**Fig. 4:**
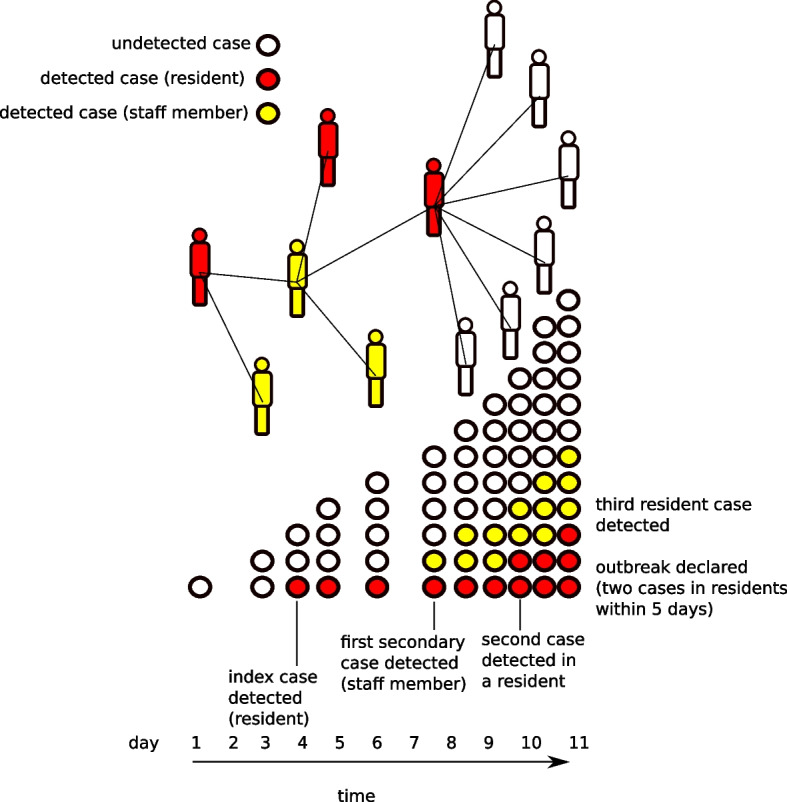
Schematic of the transmission process and outbreak declaration after detection of a specified number of cases within a given time window. Outbreaks are declared either from the detection of two cases in residents within five days, or detection of five cases in staff members within seven days

**Fig. 5:**
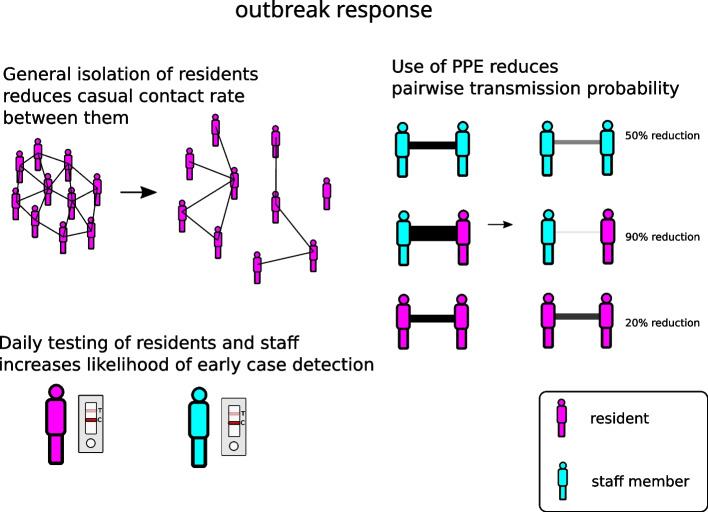
After an outbreak is declared, several different types of responses may follow. General isolation of residents reduces the rate of random contacts between them by a fixed proportion (which we vary in this study). Asymptomatic testing schedules are adjusted after the outbreak is declared, depending on the screening strategy simulated. Additionally, the deployment of PPE reduces the force of infection (pairwise transmission rate), depending on the types of individuals coming into contact, with a 90% reduction between staff and residents, a 50% reduction between staff members, and a 20% reduction between residents

The model includes asymptomatic testing of staff and residents, as well as testing on development of symptoms. Infected residents are (imperfectly) isolated from other residents upon detection, but still have contact with staff. Infected staff are furloughed for seven days upon detection, and have no further contact within a facility during the furlough period (Fig. 3). As per the Australian Government’s guidelines (dated 15th February, 2022 [20]), an outbreak is declared once two cases have been detected in residents within a five day period or five cases have been detected among staff members over a seven day period (Fig. 4). Once an outbreak has been declared, the frequency of testing of staff and residents may increase and infection control measures (e.g., use of PPE) are put in place, reducing the probabilities of transmission among staff and residents (Fig. 5). We assume differential effects of these infection control measures depending on the type of contact. For contacts between residents, we assume a relatively limited effectiveness of 20% due to factors such as lower compliance with physical distancing, inconsistent mask wearing, and limited training in the proper use of PPE. For contacts among staff members, we assume a medium effectiveness of 50% due to higher levels of compliance and training with PPE, but inconsistent infection control during contact events not involving residents. For contacts between staff members and residents, we assume the highest level of effectiveness (90%) due to high levels of compliance and training with PPE protocols (including the proper use of N95 masks) for staff members when interacting with residents. These assumptions are broadly in line with literature estimates of PPE efficacy (see, e.g., [31]). See the Supporting Information for more details related to model implementation of outbreak response measures.

### Simulated policies

We simulated the following set of policies for outbreak detection through asymptomatic screening:

1. Full asymptomatic screening: prior to outbreak declaration, staff are tested once or twice per week, for part-time and full-time employees, respectively. Residents are tested every day (noting that daily screening of residents is not a recommendation of the Australian Government’s guidelines, this represents an extreme screening scenario). During outbreaks, daily testing continues for residents, and staff are tested every day they are present as determined by the facility roster.
2. Asymptomatic screening during outbreaks: staff and residents are not subject to asymptomatic screening unless an outbreak is declared, after which they are subject to daily screening (as above).
3. No asymptomatic screening: testing is only conducted after symptom expression. No asymptomatic tests are conducted, regardless of outbreak status.
4. Unmitigated outbreaks: no testing is conducted. This means outbreaks are never declared, cases are never detected and isolated, and the transmission dynamics proceed without any mitigation measures. This ‘worst case’ scenario provides a baseline comparison against which intervention effectiveness can be assessed.

For each of the above screening policies, we vary the extent to which the resident population (regardless of case status) is socially isolated from one another following declaration of an active outbreak. Here, we present results for 0% (no general isolation), 50% (partial isolation), and 90% (stringent isolation) reductions in background contact rate. Note that residents in case isolation always have their background contact rate reduced by 90% (all scenarios). The assumption that case isolation is imperfect arises from the limitations to isolation reported in residential aged care settings due to the high prevalence of cognitive impairment and impulsive mobility behaviour, as well as the practical and legal limits to non-consensual physical restraint (see e.g., [18, 32, 33]).

**Fig. 6:**
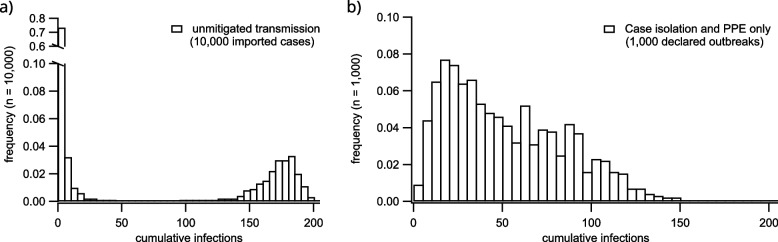
Cumulative infection distributions for simulated RACF outbreaks that are unmitigated (**a**), and which are subject to isolation of detected cases and deployment of PPE (**b**). The distribution of case numbers in (**a**) is produced over 10,000 independent stochastic simulations and includes those which are subject to early stochastic die-out. In these unmitigated scenarios, no screening takes place, no infections are detected, and no mitigation protocols are implemented. The histogram in (**b**) includes only those introductions which trigger the declaration of an outbreak, and show the case distribution over 1000 such outbreaks. More final size statistics are provided in the supporting material

This produces a set of 10 scenarios. For each screening strategy (other than the unmitigated outbreak scenario) we perform simulations until 1000 outbreaks have been simulated (the total number of simulations required for this can vary depending on the screening strategy). This produces an outbreak size distribution that is unimodal because it does not include those simulations in which transmission dies out early on and no outbreak is declared. For the unmitigated scenario (in which outbreaks are not declared), a fixed number of independent simulations are performed (10,000 instances). This produces a bimodal distribution of outbreak sizes because it includes those simulations in which transmission dies out early (Fig. 6a). We report summary statistics for the second mode (large outbreaks) to facilitate comparison between unmitigated and declared outbreaks. For each scenario, we report the following summary statistics:

- cumulative infections: the total number of infections (whether or not they are detected)
- outbreak duration: the amount of time between outbreak declaration and termination.
- peak staffing deficit: the maximum total full-time-equivalent simultaneously missing from the facility due to furlough of staff who test positive.
- cumulative residents isolated: the total number of residents placed into case isolation due to a positive test result.
- time to outbreak declaration: the time between introduction of the index case and declaration of the outbreak.
- time to first detection: the time between introduction of the index case and the first case detection.

The total number of infections is a generic measure of outbreak severity, but does not account for time-dependent properties of an outbreak. We report the outbreak duration as a measure of severity because costly and burdensome mitigation measures are in place throughout this period. The total number of residents placed into case isolation due to confirmed infection provides an additional measure of severity relevant both to apparent infection numbers and also to detrimental impacts of social isolation. The time to outbreak declaration and the time to first detection do not vary as functions of outbreak response, and provide estimates of the benefits of asymptomatic screening for early detection. The peak staffing deficit (measured in FTE) is a measure of the labour shortage (absenteeism) in the workforce due to furlough of staff. We chose to report the peak FTE deficit rather than the cumulative deficit because it provides a more intuitive measure of the logistical stress experienced within the facility due to personnel shortages. For example, if one staff member working five shifts in one week were furloughed, this would produce 1.0 FTE deficit. If one staff member working two days per week were furloughed, this would produce a 2/5 FTE deficit.

## Results

### The effects of asymptomatic screening strategies

With a reproduction ratio of $R_0 \approx 2.4$, unmitigated outbreaks can infect large proportions of the facility population. As is typical of stochastic SIR-type epidemic dynamics, outbreak sizes follow a bimodal distribution separated between those that undergo stochastic die-out during their early stages, and those that progress to large-scale epidemics. A histogram of the final size distribution for unmitigated outbreaks is provided in Fig. 6a. In contrast, even without asymptomatic testing or general isolation of residents, the use of PPE and case isolation produces a substantial drop in the distribution of outbreak size. A histogram of cumulative infection incidence for outbreaks with only PPE and case isolation in place is shown in Fig. 6b.

**Fig. 7:**
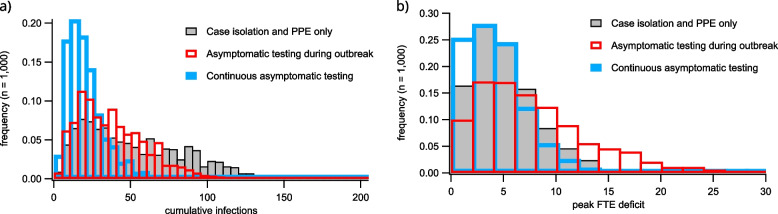
Histograms of cumulative infections (**a**) and peak FTE deficit due to furlough of staff members with confirmed infection (**b**). Grey bars correspond to scenarios employing isolation of symptomatic cases confirmed through testing and deployment of PPE after outbreak declaration. Red bars correspond to scenarios in which the declaration of outbreaks triggers asymptomatic screening of staff and residents. Blue bars correspond to scenarios in which continuous asymptomatic screening is conducted prior to outbreak declaration

Introducing asymptomatic screening after the outbreak is declared reduces infection incidence by detecting cases earlier, but increases the peak FTE deficit (Fig. 7). This is because staff who are pre-symptomatic or asymptomatic upon the initiation of the outbreak are detected within a short time window after asymptomatic screening is initiated, resulting in more staff simultaneously furloughed. In the most extreme screening scenario, with asymptomatic testing beginning before the outbreak is detected, infections are mitigated while also avoiding the increase in FTE deficit associated with outbreak response. However, such strategies require diligent screening regimes, with large numbers of tests conducted even in the absence of any detected cases.

### The effects of general isolation conditions

While effective at mitigating outbreaks, continuous asymptomatic screening of residents and staff may not be feasible under normal circumstances. The complete absence of asymptomatic screening or its implementation only during an active outbreak are more plausible scenarios, so we use them to illustrate the effects of introducing general isolation of residents. Here, we investigate to what extent the severity of outbreaks can be mitigated by general isolation conditions in combination with screening, case isolation, and PPE deployment.

**Fig. 8:**
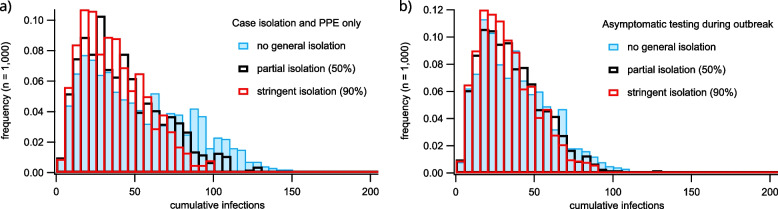
Histograms of cumulative infection numbers for outbreak scenarios involving varying levels of enforcement of a general resident isolation policy. Subfigure (**a**) shows statistics for scenarios in which outbreak response includes only isolation of symptomatic infections confirmed through testing and deployment of PPE after outbreak declaration. Subfigure (**b**) shows statistics for scenarios including asymptomatic screening after outbreak declaration. The different colored bars correspond to varying levels of isolation stringency, implemented as reductions in the rate of casual contact between residents

Our results demonstrate that, while partial and full isolation of the resident population can make a marginal difference in the absence of asymptomatic screening (Fig. 8a), it has a negligible effect when combined with asymptomatic screening during outbreaks (Fig. 8b). This somewhat surprising finding is due in part to the effectiveness of asymptomatic screening, targeted case isolation, and PPE use, and in part to a fundamental constraint to isolation of aged care residents: that residents must remain in contact with facility staff during outbreaks. Therefore, even if residents are prevented from interacting with one-another, their interactions with staff members can result in transmission. This, combined with the relatively substantial reductions in transmission from the use of PPE and the isolation of confirmed cases, leaves little to be achieved through general isolation of the resident population. For comparison with Fig. 8, frequency distributions of cumulative cases for scenarios in which all outbreak mitigation procedures (PPE, case isolation, and asymptomatic testing) are disabled are shown in the Supporting Information Figure S4. This comparison demonstrates that reducing contact between residents can mitigate against transmission and makes a substantial difference to infection totals when other response measures are not implemented.

Because we model general isolation as a reduction in the contact rate between residents, the marginal impact of general isolation is sensitive to the rate of contact between residents relative to the rate of contact involving staff members (which is not affected by general isolation). In our main results, we assume that these proportions are the same (with each rate set to an average of three per resident per day), which is consistent with empirical estimates for residential care facilities (see Discussion). However, our sensitivity analysis demonstrates that if resident-resident contact rates increase, our model produces a corresponding increase in the marginal impact of general isolation measures with lower relative increases observed for lower reproductive ratios (see Supporting Information, Figure S5). Another potential sensitivity of our model arises from the assumption that compliance with targeted case isolation is fixed at 90%. In our sensitivity analysis (see Supporting Information Figure S6), we demonstrate that our results about the marginal benefits of general isolation are robust to this assumption, as long as the condition is satisfied that general isolation compliance cannot be greater than compliance with targeted case isolation. While outbreak sizes increase when targeted isolation compliance decreases, the above condition prevents general isolation from providing a substantial additional benefit.

To supplement the findings detailed above, our full results are summarised below in Table 1.

**Table 1:** Summary statistics for model output over a range of mitigation scenarios. Medians values are shown for cumulative infections, outbreak duration, peak FTE deficit, and the cumulative number residents placed into case isolation. Next to each median value, the interquartile range is shown in parenthesis

Screening strategy	General isolation level	Cum. infections	Outbreak duration	Peak FTE deficit	Cum. residents isolated
Full asymptomatic screening	90%	14 (11)	11 (8)	3.4 (6.2)	6 (4)
Full asymptomatic screening	50%	15 (12)	12 (10)	3.6 (6.2)	6 (5)
Full asymptomatic screening	0%	17 (14.5)	14 (13)	3.6 (6)	7 (8)
Asymptomatic screening during outbreaks	90%	29 (26)	14 (7)	6.6 (11.8)	12 (11)
Asymptomatic screening during outbreaks	50%	31 (28)	15 (10.5)	6.4 (12)	13 (13)
Asymptomatic screening during outbreaks	0%	33 (32)	16 (14)	6.6 (12))	15 (17)
No asymptomatic screening	90%	32.5 (31)	16 (8)	4.4 (6.6)	9 (7)
No asymptomatic screening	50%	37 (37)	20 (12)	4.2 (7.2)	11 (12)
No asymptomatic screening	0%	44.5 (53)	24 (18)	4.4 (6.6)	15 (22)
Unmitigated outbreaks	-	$173^{\mathrm{a}}$(20)	-	-	-

$^{\mathrm{a}}$Median over all unmitigated outbreaks which produced more than 80 infections (corresponds to the second mode of the distribution shown in Fig. 6a)

## Discussion

Residential aged care environments are uniquely challenging settings for infection control and prevention [1]. The combination of high medical risk, prevalence of cognitive impairment, high service needs, and insecure workforce produces conditions in which outbreaks are more likely, infections cause more severe illness, and transmission is difficult to control. During the COVID-19 pandemic, outbreaks in these environments caused large numbers of deaths worldwide [34, 35]. Simultaneously, the measures taken to mitigate outbreaks caused disproportionate physical and mental health burdens on residents [16–19]. Trade-offs associated with resident and workforce well-being were especially pronounced before pharmaceutical interventions such as vaccines and antiviral drugs were available [12, 13]. While the availability of antivirals and vaccines have substantially reduced the pressure to apply nonpharmaceutical interventions (NPIs) in aged care environments, the prospect of future pandemics or the evolution of SARS-CoV-2 variants of concern justifies a retrospective focus on the application of NPIs.

If disease transmission modelling is to be applied effectively to inform outbreak response in such settings, models must effectively capture the semi-structured nature of the RACF environment and realistic constraints on the effectiveness of mitigation strategies. In this work, we described and demonstrated an agent-based simulator which can account for salient factors such as staff roster scheduling and non-random assignment of staff to residents, while also allowing random interactions between residents. To account for the physical, ethical, and logistical constraints arising from the uncertain behaviour of residents and from the material challenges associated with testing and PPE use, the model allows variation in compliance with scheduled testing, case isolation, and general isolation. It also allows the efficacy of PPE to vary depending on the types of individuals interacting. Another important constraint that our model accounts for is the imperfect and time-varying sensitivity of tests, which limits the degree to which isolation of detected cases can mitigate presymptomatic transmission. For realistic case detection probabilities, we implemented a previously-validated sub-model of test sensitivity as a function of time-since-infection to capture the sensitivity of tests occurring early during an infection (i.e., before expression of symptoms) [21]. With the features described above, the model was well suited to our investigation of general isolation policies and asymptomatic testing rates.

While general isolation measures may help mitigate transmission, the mental health toll produced by such measures, especially to residents near the end of life or with cognitive impairment, calls into question whether their application is beneficent. For them to be justified, it must be demonstrated that such measures provide substantial mitigation of transmission supplemental to what is achieved by those measures which may be applied at lower cost to general well-being. From this perspective, our results do not support the implementation of general isolation conditions to individuals without confirmed infection. Rather, we show that the benefits of combining (imperfect) case isolation with the deployment of PPE upon declaration of an outbreak render negligible the additional benefit achieved through general isolation measures.

Few previous modelling studies examine general isolation policies. Most recent studies focus on the role of vaccination and infection surveillance [5, 11, 36, 37], with some examining case isolation [38], cohorting [39], and furlough of staff [9]). However, our results contrast with one other study of which we are aware. The results reported by Love et al. show substantial benefits from reductions in contact rates between residents, even in combination with case isolation and other measures [30]. However, their model assumes very large differences in the level of contact between residents with and without social distancing (respectively: 2 vs. 50 potentially infectious contacts per resident, per day). The unmitigated contact rate (50 per day) is much higher than those reported by Vilches et al. (6.8 contacts per day) for Canadian care facilities [8], and Smith et al. (five per day) for a French facility [5], both of which are closer to the rate assumed in our implementation (three per day). Therefore, while their conclusions may be valid for scenarios with very high baseline contact rates, these rates are not realistic for residential aged care facilities.

Our conclusions differ also from those presented by Smith et al., which focused on optimal infection surveillance under resource-limited conditions [5]. They conclude that their results emphasize “the importance of interventions to limit contact between patients (e.g. social distancing among retirement home residents)...”, an interpretation which conflicts with our findings. However, the study by Smith et al. did not explicitly examine the effectiveness of distancing policies, so while their cautionary conclusions may be qualitatively consistent with their observations of model dynamics, these conclusions have no direct relationship to their quantitative findings. While we agree that general isolation protocols may be justified in the absence of sufficient surveillance capacity, our work illustrates that case isolation achieved through active screening may be sufficient to avoid the imposition of these potentially more harmful policies.

Asymptomatic screening is a less controversial practice than general isolation measures, but still carries many caveats. While not as acutely damaging as social isolation, the continuous imposition of physically invasive tests on staff and residents who show no signs of illness may be unjustified, especially during periods of lower community prevalence [40–42]. Furthermore, frequent asymptomatic testing may not be feasible due to resource constraints [43].

Here, we compared screening strategies employing differing levels of asymptomatic testing, and demonstrate marked improvements for scenarios in which asymptomatic screening is employed prior to declaration of an outbreak. These results are in general agreement with reports from real-world outbreaks (e.g., [44]). This result comes with the caveat that our simulations are initialised with the importation of an infectious case into the facility (infecting either a staff member or a resident). Therefore, while effective at preventing outbreaks from occurring and at staunching them earlier if they do occur, our findings are consistent with the choice to implement continuous asymptomatic screening only when community transmission presents a substantial risk of case importation. We note that while the introduction of asymptomatic screening upon outbreak declaration can decrease the final size of outbreaks, this policy naturally leads to more staff absenteeism (Fig. 7b).

### Limitations

While our model is realistic in its representation of key factors such as the facility structure, staffing schedules, within-host disease dynamics, test sensitivity, and outbreak response, in other ways the level of abstraction it applies introduces some limitations. One of these is the way in which the model simulates furlough of staff members with confirmed infection. The mechanism of case isolation for staff members is to remove them from the facility workforce for a 7-day period. While this response and its duration are realistic, we do not simulate re-assignment of rooms to the remaining staff members, an omission which could produce unrealistic reductions in network density and cause the model to over-estimate the effectiveness of furloughing staff with confirmed infection. To realistically capture labour re-distribution patterns (i.e., “surge rostering”) additional consultation is required because specific industry standards are not available. This limitation allows residents to be completely isolated due to the furlough of all staff servicing their room. We confirmed that such scenarios arise to a negligible extent (and do not occur at all in more than 90% of simulations, see Supporting Information Figure S1). Regarding the social interface of the RACF with the outside community, our model simulates only a single importation event. We do not explicitly simulate visitors to the facility, or the transmission patterns associated with staff members outside of the facility. Our model therefore implicitly assumes that visitors play a negligible role in transmission during active outbreaks, and that there is a low likelihood of multiple importations occurring prior to outbreak declaration. In scenarios with high community prevalence, multiple importation events from visitors could feasibly occur and could lead to larger numbers of presymptomatic or asymptomatic infections at the time of outbreak declaration. However, multiple importations would not alter the reproductive ratio, so we do not expect our main results regarding the marginal impact of general isolation after outbreak detection to be sensitive to the number of initial infections. Looking to future work, we note that restrictions on visitation were among the most heavily criticised policies applied to aged care facilities during COVID-19 and this subject deserves further attention focused on balancing the well-being of residents and their families with the need to prevent importation of infectious diseases. In general, while simulating the community external to the facility is beyond the scope of this study, it should be considered for future work on topics such as optimising the criteria for outbreak declaration so to avoid false alarms, or the risks produced by RACF staff working across multiple facilities. Though these topics have been studied by others (e.g., [10]), understanding the role of staffing practices in outbreak prevention and mitigation is applicable to other healthcare sectors as well and many challenges remain unaddressed [45, 46].

Incorporating the effects of vaccines and prior exposure on transmission potential is feasible with the model described here but was not implemented in this study, in which we placed the focus instead on non-pharmaceutical interventions. We made this choice in part because of the great diversity of exposure and vaccination histories that currently exist in aged care facilities, and in part because we aimed to present results with generic implications beyond COVID-19.

Finally, our choice to assume a uniform asymptomatic fraction of 33% neglects the potential for SARS-CoV-2 infection to present higher clinical fractions in older age groups. Assuming higher symptomatic proportions in the resident population due to their older age could reduce the marginal impact of asymptomatic screening. However, even with a low asymptomatic fraction, screening would still play an important role in early case identification during incubation, limiting the sensitivity of our findings to this simplifying assumption.

## Conclusion

In conclusion, our study used an agent-based simulation model to examine realistic combinations of policies for COVID-19 outbreak mitigation within residential aged care facilities. We focused our analysis on assessment of asymptomatic screening strategies, and general isolation policies for outbreak response. Our findings are consistent with the position that general isolation of residents is not justified if screening resources are sufficient for frequent surveillance, PPE is available to staff and residents, and case isolation can be conducted according to clinical practice guidelines. Furthermore, our results support the recommendation for continuous asymptomatic screening of residents and staff, with the caveats that such measures will be subject to resource constraints and analysis of case importation risk. Future work extending this study should focus on the role of hybrid immunity from vaccination and virus exposure, the facility-community interface, and the design of industry workforce practices that can help limit the likelihood of case importation.

## Supplementary Information

Supplementary Material 1.

## Data Availability

All code required for reproduction of the results reported in this study is contained in the associated GitHub repository https://github.com/cjzachreson/RACF_C19_A.

## Abbreviations

RACF: Residential aged care facility
PPE: Personal protective equipment
NPIs: Nonpharmaceutical interventions
FTE: Full-time equivalent
