# Supplementary material for: A model-based assessment of social isolation practices for COVID-19 outbreak response in residential care facilities
Source: BMC Infect Dis. 2024 Aug 29;24:880. doi: 10.1186/s12879-024-09788-x (PMC11360480; doi:10.1186/s12879-024-09788-x)
Supplement: Supplementary file 1 — Supplementary Material 1. [file 12879_2024_9788_MOESM1_ESM.pdf]

Supporting Information:
A model-based assessment of social isolation practices for COVID-19 outbreak response in residential care facilities

Cameron Zachreson*,[1] Ruarai Tobin,[2] Camelia Walker,[3] Eamon Conway,[4] Freya M Shearer,[2] Jodie McVernon,[5,6] and Nicholas Geard[1]

[1]*School of Computing and Information Systems,*
*The University of Melbourne, Parkville, Victoria, Australia**

[2]*Centre for Epidemiology and Biostatistics,*
Melbourne School of Population and Global Health,
The University of Melbourne, Parkville, Victoria, Australia

[3]*School of Mathematics and Statistics,*
The University of Melbourne, Parkville, Victoria, Australia

[4]*The Walter and Eliza Hall Institute, Melbourne, Australia*

[5]*Victorian Infectious Disease Reference Laboratory Epidemiology Unit,*
The Royal Melbourne Hospital at the Peter Doherty Institute
for Infection and Immunity, Melbourne, VIC, Australia

[6]*Department of Infectious Diseases,*
The University of Melbourne at the Peter Doherty Institute
for Infection and Immunity, Melbourne, VIC, Australia

(Dated: September 5, 2024)

Algorithm S1 Overview of the model algorithm.

```
population ← GENERATE POPULATION(facility characteristics)
network ← INITIALISE CONTACT NETWORK(population)
scenario ← SET SCENARIO CONFIGURATION(input parameters)
configuration ← (population, network, scenario)
while outbreaks < 1000 do
    initial conditions ← INITIALISE DYNAMICS(configuration)
    (outbreak declared, stats) ← TRANSMISSION(initial conditions, configuration)
    if outbreak declared then
        outbreaks ← outbreaks + 1
        push stats to ensemble output
    end if
end while
return summary statistics for all outbreaks
```

S1. DETAILED METHODS

A. Overview

The model used in this work consists of three main components: 1) a structured population contact network model based on the characteristics of an RACF environment, 2) a model of COVID-19 within-host disease progression and transmission between infected and susceptible individuals, and 3) a model of case detection and outbreak response. Pesudocode describing the overall simulation algorithm is shown in Algorithm S1 and a more detailed description of the transmission algorithm providing key details of the transmission model implementation is provided in Algorithm S2.

A list of key model parameters is provided in Tables S1, S2, and S3. The method descriptions provided in the following sections provide details of model implementation. The method description below does not contain mathematical details of the dynamic model of test sensitivity, which is described comprehensively in our previous work [1].

* cameron.zachreson@unimelb.edu.au

Algorithm S2 Outbreak simulation algorithm

```
function TRANSMISSION(initial conditions, configuration)
    outbreak declared ← false
    termination flag ← false
    while termination flag is false do
        iterate transmission dynamics:
        t ← t + Δt
        UPDATE ACTIVE INFECTIONS(population)          ▷ progress within-host model
        ----------------------------------------------------------------
        update environment:
        if new day then
            UPDATE STRUCTURED CONTACT NETWORK(day of week, network)
            TEST STAFF AND RESIDENTS(outbreak declared, scenario, population)
            UPDATE CASE ISOLATION(outbreak declared, scenario, population)
        ----------------------------------------------------------------
            outbreak response:
            if outbreak declaration conditions are met then
                outbreak declared ← true
                OUTBREAK RESPONSE(scenario)           ▷ alters testing, PPE, distancing
            end if
            if termination conditions are met then
                termination flag ← true
            end if
        end if
        ----------------------------------------------------------------
        contact sampling:
        initialise potential transmission pairs: E_I ← [ ]
        initialise aggregate weight of infectious edges: w_I ← 0
        for a ∈ infected agents do
            push edges e_a to E_I                     ▷ compile list of infectious edges
            w_I ← w_I + Σ_{e∈e_a} w_e                 ▷ sum weights of infectious edges
        end for
        sample infectious edges: E_T ⊂ E_I : p(e ∈ E_T) ∝ w_e/w_I
        push background contacts to E_T               ▷ random contacts between residents
        ----------------------------------------------------------------
        pariwise transmission:
        for e ∈ E_T do
            evaluate pairwise transmission probability
            if transmission successful then infect susceptible contact
            end if
        end for
    end while
    return outbreak declared, summary stats
end function
```

Parameters: RACF facility model		
Symbol	Description	Value
n	Number of residents	88
k	Number of facility staff	121
p_{med}	Prop. of medically-trained staff	0.3
p_{needs}	Prop. of high-needs residents	0
p_{shared}	Prop. of double-occupancy rooms	0
$p_{\min}$	minimum fraction of staff present each day in baseline roster	0.2
p_5	prop. of staff working 5 days per week	0.26
p_3	prop. of staff working 3 days per week	0.36
p_2	prop. of staff working 2 days per week	0.38
k_{gen}	number of general staff assigned to each room per day	4
k_{med}	number of medical staff assigned to each room per day	1
Structured contact network		
c	mean number of contacts per resident, per day	3.0
$\lambda_{(\mathrm{i})}$	mean total contact rate (contacts per day)	nc
w_e	sampling weight for each edge	see Table S4
Unstructured contact network		
$\lambda_{(\mathrm{ii})}$	mean number of background contacts per resident per day (baseline, no active outbreak)	3.0

TABLE S1. Summary of key model parameters related to population structure. For additional details regarding the facility model and contact networks see the corresponding sections below.

.

Parameters: COVID-19 transmission model		
Symbol	Description	Value
κ	global transmission scalar	0.2
R_0	mean reproductive ratio	≈ 2.4
δ	dispersion parameter of secondary case distribution	0.1
β_{max}	random variable determining an individual's peak infectiousness	Gamma (shape = δ, scale = κ/δ)
T_{inc}	mean incubation period	5.5 days
t_{inc}	random variable determining an individual's incubation period	Lognormal ($\mu = 1.62$, $\sigma = 0.418$)
T_{rec}	mean recovery period	7.5 days
t_{rec}	an individual's recovery period	Uniform(5, 10)
p_{asymp}	mean proportion of asymptomatic infections	0.33

TABLE S2. Summary of key model parameters. For additional details on the COVID-19 transmission model, see the relevant section below and our previous work [1].

Parameters: Outbreak detection and response		
Symbol	Description	Value
ρ_{max}	peak test sensitivity<br>(see [1] and [2] for further details)	≈ 0.83
$\Delta_{\text{min}}^{\text{staff}}$	minimum time (in days)<br>between asymptomatic tests<br>for staff members (no active outbreak)	3
$p_{\text{test}}^{\text{resident}}$	probability per day of an asymptomatic<br>resident testing for infection	0 or 1<br>(depends on scenario)
$p_{\text{test}}^{\text{symptoms}}$	probability of testing if symptomatic<br>(note: staff members are only tested if present)	1.0
t_{furlough}	duration of furlough period for staff who test positive	7 days
t_{iso}	duration of case isolation period for residents who test positive	7 days
p_{iso}	case isolation efficacy	0.9
L	general isolation efficacy	0.9, 0.5, or 0<br>(depends on scenario)
η_{ab}	efficacy of infection control (e.g., PPE)	0.2, 0.5, or 0.9<br>(depends on context<br>see Table 5)

TABLE S3. Summary of key model parameters. For additional details regarding the outbreak response model (including screening strategies, criteria for declaring outbreaks and criteria for declaring outbreaks to be over) see the corresponding sections below.

.

B. Facility Characteristics

In our model, a residential aged care facility is described by the following characteristics:

- number of residents n
- number of staff k
- proportion of staff who are medically trained p_{med}
- proportion of residents with high needs p_{needs}
- proportion of shared rooms p_{shared}

For the results reported in this study, we simulated a single facility with the values for these parameters listed in Table S1. The number of rooms is determined by the number of residents and the proportion of shared rooms, and assumes that all shared rooms are double-occupancy (Note that in this work we assume all rooms are single-occupancy).

Assignment of staff to a work roster follows the data on full-time-equivalent (FTE) and number of sector employees described in the 2020 census of Australian aged care facilities [3]. Rooms are allocated to staff members based on their role (medical or general staff) and are allocated to ensure all rooms are serviced while distributing the number of room assignments as evenly as possible among the workers available on each day of the roster period (1-week). More details are provided below:

1. **Staff types:** All staff are either Personal Care Workers (PCWs), or nurses and allied health professionals (medical staff). These all fall under the category of "direct care staff". In other words, we do not model the activities of "ancillary" staff (i.e., cooking, laundry, cleaning).

2. **Medically trained staff:** We assume that 30% of the aged care workforce is medically trained (nurses or allied healthcare). This assumption is based on the headcount numbers in the 2020 Aged Care Workforce Census Table 2.2 [3]

3. **Minimum staffing (under normal conditions):** Under normal conditions (no active outbreak) the number of staff present at the facility may not be less than 1/5

the total headcount (i.e., if a facility has 100 PCWs, a roster will not be simulated that has fewer than 20 of them present on any given day). This constraint is applied separately to the medical staff and PCWs, and exists to ensure that rosters are not generated with unrealistically low numbers of staff present.

4. **Staff roster:** The staff roster repeats weekly, and is generated by assuming 26% of staff work 5 days/wk, 36% work three days per week, and 38% work two days per week. This is derived from the headcount totals and FTE (see the 2020 Aged Care Census report Table 2.2 [3]).

5. **Assignment of rooms to staff:** On a given day, each room of a facility is serviced by exactly 4 PCWs and 1 medical staff member. The allocation of rooms to staff members attempts to produce uniform labour distribution (though the specific constraints applied to individual facilities require some relaxation of this rule). This is done to ensure that all rooms are serviced by multiple staff members, and that individual staff members are not allocated a disproportionate number of rooms.

6. **Assignment of rooms to residents:** Each resident is assigned to one room.

7. **Consistency in the assignment of staff to sets of rooms:** To the extent allowed by the intermittent work schedule defined by the roster (and the staffing requirement of each room), staff are assigned the same set of rooms on each day they are present at the facility. This is designed to simulate the tendency for staff to be assigned consistently to the same locations, and can be adjusted to produce increased levels of randomness in room assignments from day to day.

C. within-host model of disease progression

The within host model of disease transmission features infectiousness that increases from the time of infection, peaking closely before symptom onset, and then declining until recovery. Crucially, we assume an over-dispersed distribution of peak infectiousness values which corresponds to a negative binomial secondary case distribution, if a sufficiently large number of susceptible contacts exist.

Each infected individual's infectiousness varies with the time since infection τ. The trajectory is a 3-part piece-wise function described by an initial increase, a brief plateau (spanning the end of the incubation period), and a decline towards recovery:

$$\beta(\tau) = \begin{cases} \frac{\beta_{\max}}{V_{\max}}\Big[\exp(k_1\tau) - 1\Big] & \tau \leq t_{\text{inc}} - T_p \\ \beta_{\max} & t_{\text{inc}} - T_p \leq \tau < t_{\text{inc}} \\ \beta_{\max}\Big[\exp\Big(k_2[\tau - t_{\text{inc}}]\Big) - [V_{\max}]^{-1}\Big] & \tau \geq t_{\text{inc}} \end{cases}, \tag{S1}$$

where t_{inc} is the incubation period of the individual, T_p is the duration of the infectiousness plateau (set equal to $0.1t_{\text{inc}}$), and $\beta_{\max}$ is the maximum infectiousness of that individual. The parameter $V_{\max}$ is controls the shape of the growth curve (smaller values of $V_{\max}$ produce broader growth and decay functions, higher values produce steeper growth and decay). The rate parameters k_1 and k_2 are determined by the value of $V_{\max}$, and the duration of incubation and post-incubation periods (which are sampled for each infected individual):

$$k_1 = \ln(V_{\max})\ [t_{\text{inc}} - T_p]^{-1}\,, \tag{S2}$$

and

$$k_2 = \ln\Big([V_{\max}]^{-1}\Big)\ t_{\text{r}}^{-1}\,, \tag{S3}$$

where t_r is the time between symptom onset and recovery, which is drawn uniformly at random from the range [5d, 10d], to capture a plausible range for the duration of infectiousness after symptom onset with a mean of $T_{\text{rec}} = 7.5$ days.

In the individual-level model of infectiousness described above, the incubation period plays a central role in determining the dynamics of infectiousness. For each individual, these are drawn at random from a lognormal distribution with log-mean $\mu_{inc} = 1.62$ and $\sigma = 0.418$ (for a mean incubation period of $T_{\text{inc}} = 5.5$ days). For asymptomatic infections, the time-dependent infectiousness trajectory is computed identically as for those which do express symptoms. The only difference is that symptoms cannot be observed after incubation and symptomatic screening for case detection is not conducted. We simulate a mean asymptomatic fraction of 33% using a probabilistic implementation: at the moment of infection, an individual will be labeled as asymptomatic with a probability of $p_{\text{asymp}} = 0.33$. This asymp-

tomatic fraction is in line with global estimates but we do not account for heterogeneity by age [4–7].

To produce the over-dispersed secondary case distribution shown in Figure **??**, the individual-level parameter β_{max} is drawn from a Gamma distribution with shape parameter $k_{\text{shape}} = 0.1$ and scale parameter $s = \kappa/k_{\text{shape}}$, where the mean κ globally scales the transmission rate and reproductive ratio. This implementation produces a negative-binomial secondary case distribution with dispersion $\delta = 0.1$, equivalent to the shape parameter of the Gamma distribution for β_{max}.

Additional details of the model of within-host infectiousness, including calibration, can be found in our previous work [1].

D. calibration of the reproductive ratio

To calibrate the basic reproductive ratio, we systematically varied the global transmission scalar κ. For each value of κ, we simulated an ensemble of 1000 index cases and recorded the number of secondary cases produced for each instance. The mean of each ensemble provided an estimate of the reproductive ratio for the corresponding value of κ. Figures describing the calibration of $R_0(\kappa)$ as well as final size statistics for reach value of κ are shown in Figure S2 and Figure S3. Based on this calibration we selected $\kappa = 0.2$ to produce a reproductive ratio $R_0 \approx 2.4$. To ensure index cases were selected commensurate with their relative likelihood of being infected during an outbreak, index case selection was weighted based on the sum of all contact edge weights associated with each agent (see below for more details about the contact network specifications).

E. test sensitivity

The time-dependent test sensitivity function is derived from a study that performed prospective serial testing of healthcare workers [2] and follows a similar trajectory to infectiousness, with sensitivity peaking just before symptom onset and a gradual decline afterwards. Test sensitivity of rapid antigen tests is assumed to peak at approx. 83% on average (though this varies from person to person). Extensive details about the specifics of the test

sensitivity model used here can be found in our previous work [1]. We assume tests are 100% specific (no false positives).

F. network model of disease transmission

To simulate disease transmission within the facility population, we employ a contact network model. This model has two components (i) a structured network built to represent potential co-location dynamics within the rooms of the facility and (ii) a homogeneous network of random interactions between residents representing unstructured contact due to (e.g.) group activities, shared meals, or casual social interactions.

1. component (i): structured contact network

Component (i) of the transmission network is a dynamic weighted multigraph (multiple edges may exist between two nodes) that includes both staff members and residents as nodes. The edges in the network connect staff members who are assigned to service the same room on the same day, residents to their roommates, and residents to the staff members who service their rooms. Figure **??** provides a schematic description of how this part of the network is generated, based on the assignments of residents and staff to rooms. The network topology of component (i) is dynamic, changing for each day of the simulation based on which staff are assigned to which rooms. Furlough of staff members who test positive also changes the network topology by removing all connections with furloughed staff members. These connections are reinstated after the 7-day furlough period. The edges in component (i) are weighted, to account for the relative likelihood that a given edge is sampled during the transmission simulations (described below). Specifically, edge weights depend pairwise on the types of agents they connect as shown in Table S4. For staff members who are assigned more than one of the same rooms, an edge exists between them for each room they share.

type of interaction	weight
worker → worker	1.0 (baseline)
resident → resident (different room)	1.0 (baseline)
worker → resident (typical needs level)	2.0
worker → resident (high needs)	6.0
resident → resident (same room)	10.0

TABLE S4. Sampling weights used for the edges of the structured contact network component of the transmission model.

2. component (ii): random contacts between residents

Component (ii) of the transmission network is implemented implicitly as a weighted mixing network. Residents contact one another at a constant rate, implemented as a Poisson-distributed number of contacts over discrete time intervals. Weights are introduced for non-uniform edge sampling in order to account for the reduced likelihood of contacting another resident who is subject to general isolation or case isolation (see below).

3. contact sampling

In each discrete simulation timestep (of duration $\Delta t = 0.1$ day), pairwise transmission dynamics are evaluated between infected individuals, and sampled subsets of their potential contacts. This sampling process is split into components (i) and (ii) as described above. Here, we describe the details of our contact sampling algorithm for components (i) and (ii). We note that the network topology of component (i) is a function both of the day of the staff roster cycle and of the set of staff who are furloughed and not present at the facility. In the description below, we omit notation associated with these two aspects of network structure dynamics, in order to clearly denote the process used to sample infectious contacts from the set allowed by network structure at any time t.

For component (i), sampling depends on the total contact rate for component (i) $\lambda_{(\mathrm{i})}$, the aggregate weight of edges in component (i) $w_{(\mathrm{i})}$, the set of edges connected to infected

individuals $\{E_I\}$, and their aggregate weight w_I. The mean number of potentially infectious edges to evaluate in a timestep is then given as:

$$\lambda_I = \lambda_{(\text{i})} \frac{w_I}{w_{(\text{i})}} \Delta t \,, \tag{S4}$$

i.e., the expected total number of contacts during the timestep multiplied by the proportion of infected edges. The number of potentially infectious contacts from component (i) $c_{(\text{i})}(t)$ sampled for a given timestep is then drawn from a Poisson distribution:

$$c_{(\text{i})}(t) \sim \text{Poisson}(\lambda_I) \,, \tag{S5}$$

and the $c_{(\text{i})}(t)$ contacts on which transmission is evaluated are selected at random (with replacement) from the set $\{E_I\}$ with sample probability of each edge $e \in \{E_I\}$ equal to w_e/w_I where w_e is the weight of edge e. This produces a set of edges sampled from component (i), $\{E_{(\text{i})}\}$.

For component (ii), the unstructured contacts between residents, sampling is implemented by iterating through infected residents and sampling contacts from the set of other residents in the facility population. The sampling algorithm depends on the baseline rate of casual contact *per resident* for component (ii) $\lambda_{(\text{ii})}$, the general isolation level L, the effectiveness of case isolation p_{iso}, the set of residents $\{r\}$ who are infected at time t, and their isolation status $\{\theta\}$ (which takes a value of $\theta_i = 1$ if resident r_i is isolated). For each infected resident r_i, the mean number of contacts sampled is given as:

$$\lambda(r_i) = \lambda_{(\text{ii})}(1 - \alpha_i)\Delta t \,, \tag{S6}$$

where

$$\alpha_i = p_{\text{iso}}\theta_i + L(1 - \theta_i) \,, \tag{S7}$$

to account for the reduction in contact produced by case isolation or general isolation of resident r_i (note that case isolation effects supersede those of general isolation, but the two effects do not multiply). The number of potentially infectious contacts from component (ii)

contributed by resident r_i, $c_{\text{(ii)}}(r_i)$, is sampled from a Poisson distribution:

$$c_{\text{(ii)}}(r_i) \sim \text{Poisson}(\lambda(r_i))\,. \tag{S8}$$

These $c_{\text{(ii)}}(r_i)$ edges are selected at random from the set of all other residents $\{r_{k\neq i}\}$ with sample probability equal to:

$$\frac{(1-\alpha_k)}{\sum_{r_i\in\{r\}}(1-\alpha_i)}\,, \tag{S9}$$

which, compiled over all infected residents produces the set of potentially infectious edges contributed by component (ii): $E_{\text{(ii)}}$. The union of infectious edges from components (i) and (ii) gives the full set of edges to evaluate for disease transmission at time t: $\{E_T\} = \{E_{\text{(i)}}\} \cup \{E_{\text{(ii)}}\}$.

G. pairwise disease transmission

Once the set of potentially infectious contacts $\{E_T\}$ is determined through sampling, the probability of infection between infected individual a and individual b (who may or may not be infected) is computed for each edge as as:

$$p_{\text{trans}}(a,b,\tau) = [1-\exp(-\beta_a(\tau)\eta_{ab})]S_b\,, \tag{S10}$$

where β_a is the force of infection produced by the infected agent a, τ is the time since individual a was initially infected, η_{ab} is a reduction factor for PPE effects (which depend on whether individuals a and b are residents or staff members), and S_b takes a value of 0 if individual b is infected or recovered and 1 if they are susceptible. Note that the role of the timestep Δt is not included in Equation S10 because it is accounted for in the contact rates described in Equations S4 and S6. The transmission rate β_a changes as a function of time since infection, as described by our within-host model of disease transmission which accounts for dispersion of infectiousness among infected individuals, and the timing of transmission as described in detail in our previous work [1].

H. implementation of interventions and PPE deployment

The assumptions listed below with respect to screening for cases and response to outbreaks were informed by the advice detailed in the Australian Government Department of Health document entitled "COVID-19 Outbreaks in Residential Care Facilities, Communicable Disease Network Australia, National Guidelines for the Prevention, Control and Public Health Management of COVID-19 Outbreaks in Residential Care Facilities" dated February 15th, 2022 [8]. [Note: the Australian Government recommendations have been modified since this study was conducted, the version of the guidelines we used to inform our model is included as Supporting Material to ensure continued access.]

1. Staff screening (no active outbreak). Staff are tested periodically, with no more than two tests scheduled per roster period (weekly) and a minimum of $\Delta_{\text{min}}^{\text{staff}} = 3$ days between subsequent tests. This means full-time workers are scheduled for two tests per week and part time workers are scheduled for one test per week.

2. Resident screening (no active outbreak). Residents are not subject to scheduled testing, but have a baseline probability $p_{\text{test}}^{\text{resident}}$ of being tested on any given day which varies depending on the screening scenario.

3. Screening (active outbreak). During an active outbreak, staff are scheduled for tests on each day they are present at the facility. The probability of a resident being tested on a given day is adjusted as well, depending on the screening strategy implemented.

4. Screening (symptom expression). Any individual who expresses symptoms (regardless of whether or not an outbreak has been declared) is tested if they are present at the facility. If they test negative, they are not required to isolate. They will be tested again on each day they express symptoms (i.e., a single negative test is not sufficient to avoid additional screening if the individual is symptomatic).

5. Efficacy of infection control measures. After outbreaks are declared, infection control measures are put into place. These reduce the probability of transmission between different types of individuals according the the following efficacy assumptions:

type of interaction	reduction in force of infection (η_{ab})
resident → resident	0.2
worker → worker	0.5
worker → resident	0.9

TABLE S5. Efficacy of infection control measures (e.g., PPE), which depends on the types of individuals interacting (η_{ab}).

These choices reflect the assumption that residents use PPE and employ protective behaviour only on a discretionary basis. Staff, on the other hand, are assumed to utilise PPE to a greater extent (as per best-practice guidelines), and to almost always use PPE during interactions with residents.

6. Reduction in background contact frequency between residents during active outbreaks. The model assumes that background contact rates between residents fall by a fixed proportion during active outbreaks to simulate physical distancing, cancellation of group activities, limitations to use of communal areas (e.g., avoiding group meals), or in-room isolation policies.

7. Throughout, we assume a 90% reduction in background contact frequency for residents who are isolated after testing positive ($p_{\text{iso}} = 0.9$). This reflects the imperfect capacity to isolate a resident to their room at all times. [We note here that contact rates with staff and with roommates are unaffected by resident case isolation]. Case isolation lasts for a fixed period of $t_{\text{iso}} = 7$ days.

8. Furlough of staff members with confirmed infection continues for $t_{\text{furlough}} = 7$ days after the day of detection. For the seven day period, a furloughed staff member is removed from the model (their connections are removed from the transmission network). We do not simulate re-distribution of room assignments upon furlough of staff, rather, we assume that all network connections not involving furloughed staff members remain unchanged. This produces a small probability that residents will become completely isolated from all staff members servicing their rooms (an unrealistic condition). For quality control, we quantified this probability for the scenario producing the highest

FTE deficit (this occurs when asymptomatic screening is implemented upon outbreak declaration, with general isolation level set to zero, see Table ??). The analysis summarised in Figure S1 demonstrates negligible levels: for a sample of 1000 simulated outbreaks, 90.1% showed zero resident-days of isolation due to staff furlough, 5.5% of simulated outbreaks produced one resident-day of isolation due to staff furlough, 3.5% produced two to five resident-days, and 0.2% produced six to nine resident-days of isolation due to staff furlough.

9. Declaring an active outbreak: For a facility to declare an active outbreak, one of two criteria must be met: (i) two new resident cases within 5 days or (ii) five new cases in staff within seven days as per the Australian Government guidelines [8].

10. Declaring an active outbreak over: If no new resident cases have been detected within seven days, the outbreak is considered to be over, and infection control measures cease.

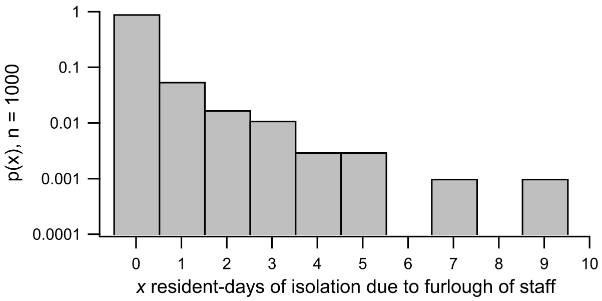

FIG. S1. Frequency of resident-days of isolation due to furlough of staff members over 1000 outbreaks. For these simulations, the condition producing the maximum FTE deficit was selected (asymptomatic screening during outbreaks, with 0% general isolation level, see Table ??). Over the ensemble of 1000 simulated outbreaks, 90.1% produced zero, 5.5% produced one, 3.5% produced two to five, and 0.2% produced six to nine resident-days of isolation due to staff furlough.

I. vaccine-derived protection

The effects of vaccination were not the focus of this study and are not explicitly simulated. However, the agent-based model implementation provided in the linked repository is capable of simulating vaccine-derived reductions in transmission. The vaccination model implemented there links vaccine-derived protection to an individually-assigned correlate of

protection, using the logit-normal vaccine efficacy model described in our previous work [9], with default parameters corresponding to the immune-derived protection imparted against infection with the ancestral variants of SARS-CoV-2, after previous infection. These parameters can be adjusted to account for the effects of vaccination against different variants.

S2. CALIBRATION OF R_0

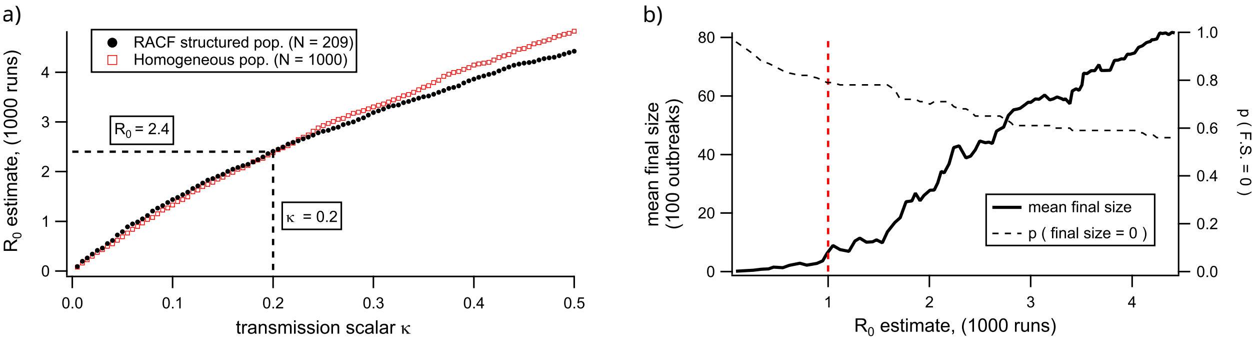

FIG. S2. Reproductive ratios (a) and mean final size (b) as a function of the global transmission scalar κ. The reproductive ratios in (a) are computed over 1000 independent index case simulations for the RACF population used for the main results (black dots) and also for a homogeneous population of 1000 individuals (red squares). The R_0 value used in our study is shown by the dashed lines in (a). The final size statistics in (b) are computed over 100 introductions and include those which do not meet the criteria for outbreak declaration (i.e., they include those simulations which are subject to stochastic die-out). The dashed trace in (b) indicates the probability that no secondary cases will be generated, for each value of κ [right y axis in (b)].

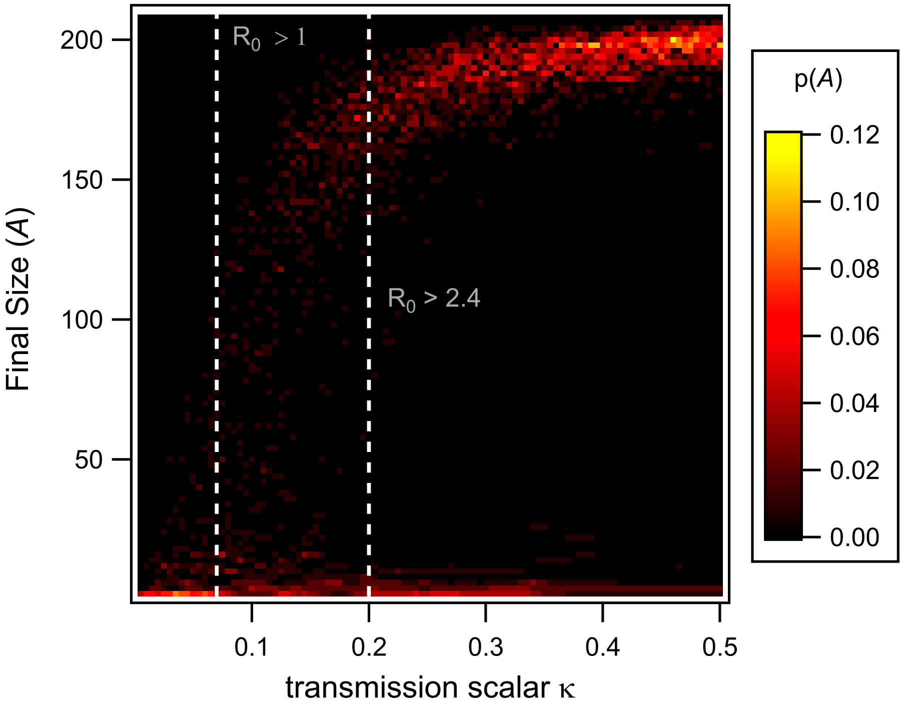

FIG. S3. Heat map of final size probabilities as a function of the global transmission scalar κ. Because we employ an overdispersed secondary case distribution, this heatmap excludes $p(A = 0)$ to facilitate visualisation (note that $p(A = 0)$ is shown in Figure S2b). Dashed lines correspond to the theoretical critical threshold $R_0 = 1$ and the reproductive ratio used for our main results $R_0 \approx 2.4$.

S3. GENERAL ISOLATION EFFECTS WITHOUT CASE ISOLATION OR PPE

Figure S4 shows final size distributions (cumulative infections) for counterfactual scenarios in which general isolation is the only available intervention (no case isolation, furlough, or PPE).

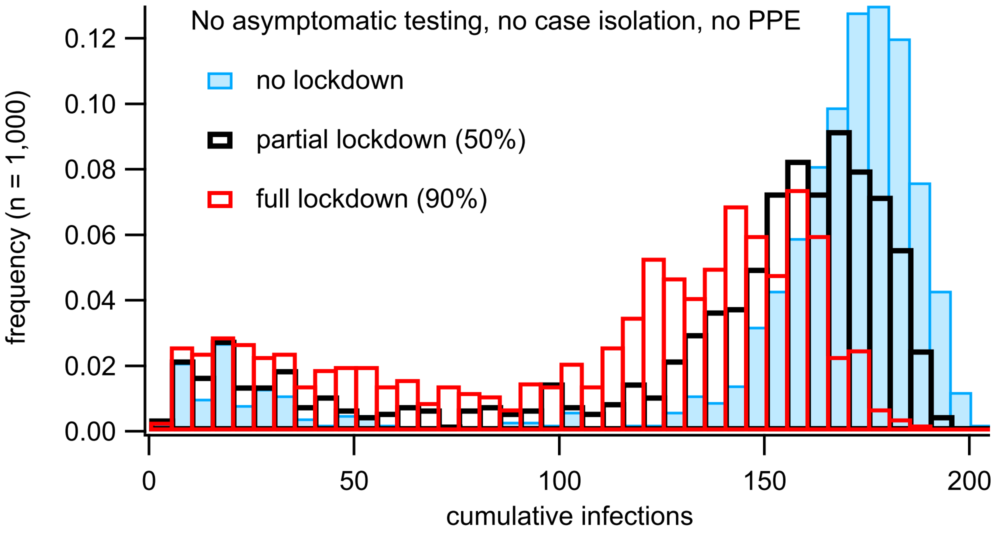

FIG. S4. Effects of restricting resident-resident contact rates during outbreaks. Frequency distributions of cumulative infection numbers for each of 1000 outbreak simulations are shown for unmitigated outbreaks (blue bars), partial isolation of residents (contact rates between residents reduced by 50%, black bars), and stringent isolation (contact rates between residents reduced by 90%, red bars). For these simulations, all outbreak response measures other than general isolation of residents were disabled (i.e., PPE, case isolation, furlough of staff members, and asymptomatic testing were not implemented).

S4. SENSITIVITY TO BACKGROUND CONTACT RATE $\lambda_{(\mathrm{ii})}$ AND CASE ISOLATION COMPLIANCE

Our main results on the marginal impact of general isolation conditions are sensitive to the rate of contact between residents (reduced through general isolation) relative to the rate of contact in the structured network (unaffected by general isolation). To investigate this sensitivity, we fix the number of structured contacts per resident fixed at $c = 3.0$, and compute outbreak final size while systematically varying the unstructured contact rate per resident ($\lambda_{(\mathrm{ii})}$). For fair comparisons between scenarios, we fix the reproductive ratio R_0 by jointly varying κ to control the transmission rate for each value of $\lambda_{(\mathrm{ii})}$. The results in Figure S5 demonstrate that our primary results hold when the ratio $\lambda_{(\mathrm{ii})}/c$ is near one. Even for larger values of $\lambda_{(\mathrm{ii})}/c$, the marginal impact of general isolation decreases substantially as the reproductive ratio decreases (Figure S5).

The sensitivity of our main results to the level of compliance with case isolation is shown in Figure S6, which demonstrates that marginal impacts of general isolation are not sensitive to assumptions regarding case isolation compliance levels. The robustness of this result depends on the realistic assumption that general isolation compliance is bounded from above (cannot be greater than) compliance with targeted case isolation.

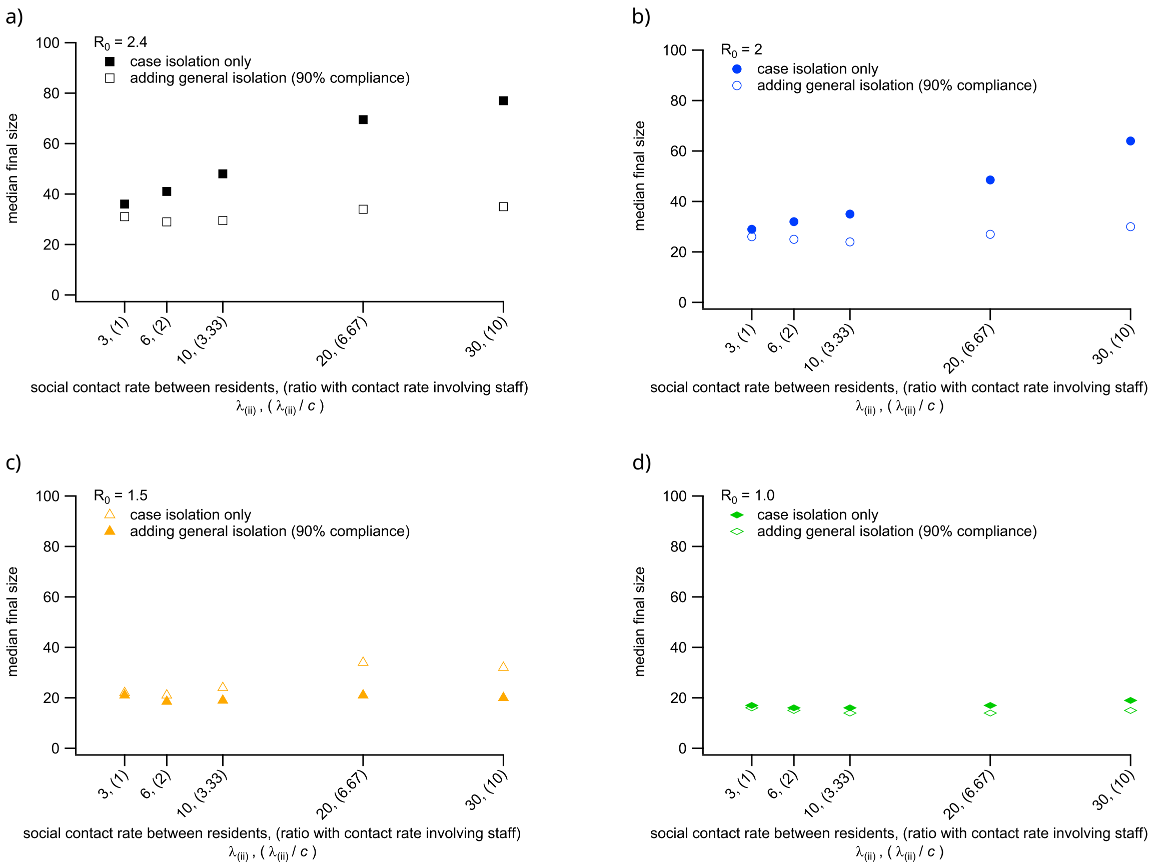

FIG. S5. Sensitivity analysis of background contact rate on the marginal impact of general isolation. Sample medians (n = 1000 outbreaks) are shown as functions of the ratio of the unstructured contact rate per resident $\lambda_{(ii)}/c$, where c is the rate of contact associated with staff members and is unaffected by general isolation measures for residents. Results are shown for different values of the reproductive ratio R_0 (a-d), with and without the application of general isolation for outbreak control. Marginal impacts of general isolation increase with R_0 and $\lambda_{(ii)}$.

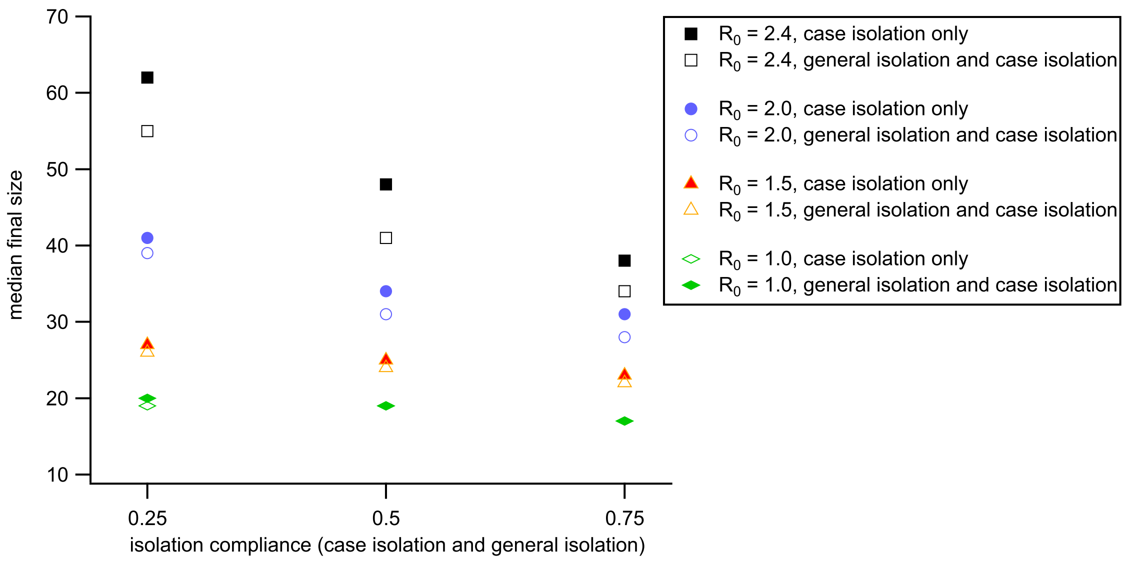

FIG. S6. Sensitivity analysis of case isolation compliance on the marginal impact of general isolation. Sample medians (n = 1000 outbreaks) are shown as functions of case isolation compliance rates for residents. Results are shown for four different values of R_0 with and without the application of general isolation for outbreak control. Here, we assume compliance with general isolation is equal to the compliance with case isolation.

S5. REFERENCES

[1] Zachreson C, Shearer FM, Price DJ, Lydeamore MJ, McVernon J, McCaw J, et al. COVID-19 in low-tolerance border quarantine systems: Impact of the Delta variant of SARS-CoV-2. Science Advances. 2022;8(14):eabm3624. Available from: https://www.science.org/doi/abs/10.1126/sciadv.abm3624.

[2] Hellewell J, Russell TW, Beale R, Kelly G, Houlihan C, Nastouli E, et al. Estimating the effectiveness of routine asymptomatic PCR testing at different frequencies for the detection of SARS-CoV-2 infections. BMC medicine. 2021;19:1-10.

[3] Department of Health. 2020 Aged Care Workforce Census Report. Australian Government; 2020.

[4] Shang W, Kang L, Cao G, Wang Y, Gao P, Liu J, et al. Percentage of asymptomatic infections among SARS-CoV-2 omicron variant-positive individuals: a systematic review and meta-analysis. Vaccines. 2022;10(7):1049.

[5] Ma Q, Liu J, Liu Q, Kang L, Liu R, Jing W, et al. Global percentage of asymptomatic SARS-CoV-2 infections among the tested population and individuals with confirmed COVID-19 diagnosis: a systematic review and meta-analysis. JAMA network open. 2021;4(12):e2137257-7.

[6] Oran DP, Topol EJ. The proportion of SARS-CoV-2 infections that are asymptomatic: a systematic review. Annals of internal medicine. 2021;174(5):655-62.

[7] Sah P, Fitzpatrick MC, Zimmer CF, Abdollahi E, Juden-Kelly L, Moghadas SM, et al. Asymptomatic SARS-CoV-2 infection: A systematic review and meta-analysis. Proceedings of the National Academy of Sciences. 2021;118(34):e2109229118.

[8] Communicable Disases Network Australia. COVID-19 Outbreaks in Residential Care Facilities, Communicable Diseases Network Australia National Guidelines for the Prevention, Control and Public Health Management of COVID-19 Outbreaks in Residential Care Facilities. Australian Government Departmnt of Health and Aged Care; 2022.

[9] Zachreson C, Tobin R, Szanyi J, Walker C, Cromer D, Shearer FM, et al. Individual variation in vaccine immune response can produce bimodal distributions of protection. Vaccine. 2023.
